# Determinants of severe acute malnutrition among under five children in rural Enebsie Sarmidr District, East Gojjam Zone, North West Ethiopia, 2016

**DOI:** 10.1186/s40795-018-0211-5

**Published:** 2018-02-09

**Authors:** Abate Awoke, Mulatu Ayana, Tenaw Gualu

**Affiliations:** 1 East Gojjam Zone Health Department, Debre Markos, Ethiopia; 2 Department of Public Health, College of Health Sciences, Debre Markos University, Debre Markos, Ethiopia; 3 Department of Nursing, College of Health Sciences, Debre Markos University, P.O.BOX: 269, Debre Markos, Ethiopia

**Keywords:** Determinants, Severe acute malnutrition, Under five children, Rural district, Enebsie Sarmidr, Ethiopia

## Abstract

**Background:**

Severe acute malnutrition is one of the major public health problems in developing countries having a devastating effect on the lives of many children under 5 years of age. In Ethiopia, there has been isolated studies conducted on malnutrition with no study attempting to identify the determinants of severe acute malnutrition in the rural district of Enebsie Sarmidr.This study intends to identify the determinants of severe acute malnutrition in rural district located in North West Ethiopia.

**Methods:**

A Community based un matched case -control study was carried on 311 (64 cases and 247 controls) children aged between birth–59 months with their respective mothers or care takers from March 1–30/ 2016. Odds Ratio along with 95% confidence interval was estimated to identify determinants of severe acute malnutrition using the multivariable logistic regression.

**Results:**

The response rate was 97.8%. Severe acute malnutrition was significantly associated with age groups birth-24 months (AOR = 2.64, 95% CI 1.17–5.95), late initiation of breast feeding greater than an hour after birth (AOR = 4.26, 95% CI 1.74–10.42), nonexclusive breast feeding (AOR =5.81, 95% CI 1.80–18.79), diarrheal disease in the preceding 2 weeks before SAM (AOR = 7.98, 95% CI 2.57–24.74), febrile illnesses preceding 2 weeks before SAM (AOR = 2.87 95% CI 1.13–7.63), decreased or maintained mealing of the mother compared to the regular during pregnancy or lactation (AOR = 8.15, 95% CI 3.70–17.98) and birth interval less than 2 years (AOR = 3.34, 95% CI 1.55–7.20) after controlling other variables effect.

**Conclusion:**

A child’s age, late initiation of breast feeding, nonexclusive breast feeding, diarrheal diseases and febrile illnesses preceding 2 weeks before SAM, decreased or maintained mealing compared to the regular during pregnancy and lactating of the mother and narrow birth interval were identified as determinants of SAM. Therefore, collaborative efforts are needed to improve promotion of better child caring practices specifically, child and maternal feeding practices and prevention and treatment of acute illnesses.

## Background

Malnutrition, globally, is linked directly or indirectly to death or disability. In 2015, wasting related to malnutrition threatened the lives of 50 million children under 5 years of age, globally, of which 3.5 million will succumb [1].

In Africa 14.1.million cases of wasting have been identified with one third or 4.3 million categorized as severe wasting. Eastern Africa has,also, identified 6.6% of children under 5 years of age as wasted due to malnutrition [2]. Ethiopia has the region’s highest rate of wasting, or acute malnutrition with 9% identified as wasted and 33.3% severely malnourished [3]. The prevalence of malnutrition ranges from 1.4% in Addis Ababa to 10% in Somali with a further distinction of higher prevalence of severe malnourishment. In many health facility, deaths are attributed to malnutrition; one study estimated 57% [4], and another stated 20% that further identified severe acute malnourishment as the cause of death [5, 6].

Different reasons can be considered for possible risk factors for severe acute malnutrition (SAM) around the world including Ethiopia. However, with the problem higher in developing countries like Ethiopia and the risk factors, may be seasonal or pervasive. Maternal, childhood or family based problems could be a determinant of nutrition and by contrast malnutrition. The variance is then related to the geographical location, the time of year, and the sex of the child [7, 8]. For example, differences are observed by gender and urban-rural residence; wasting affects more boys (11.1%) compared with girls (8.2%) and more children in rural areas (10.2%) compared with urban areas (5.7%) [6].

Over the 30 years, Ethiopia has been implementing different programs to overcome SAM with little effect as the diagnosis remains high and the prevalence has been increasing. As a result, identifying specific determinants of SAM in children under 5 years of age will assist in directing significant investments to overcome the problem [9].

### Objectives

- To identify determinants of severe acute malnutrition among rural district of children under 5 years of age living in Enebsie Sarmidr district, North West Ethiopia, 2016.

## Methods

### Study area and period

The study was conducted in the rural district of Enebsie Sarmidr, East Gojjam Zone, North West Ethiopia from March 1–30/2016. Enebsie Sarmidr district is located in East Gojjam Zone which is 364 km (km) far from Addis Ababa the capital city of Ethiopia, 182 km far from Bahir Dar the capital town of Amhara region and 194 km away from Debre Markos the capital town of East Gojjam Zone. The district has one district public hospital, seven public health centers, two medium private clinics and two urban and 31 rural health posts. Six health centers has stabilizing centers for complicated SAM cases management. The study area selection was done after review of the district’s annual report, as well there was paucity of research on SAM in this district.

### Study design

A community based unmatched case control study design was employed to identify the risk factors of severe acute malnutrition among children under 5 years of age. Unmatched case control study was used because the number of the cases were small when compared to the number of controls. This study design was taken to determine the risk factors of SAM at the single point, case identification, followed by data collection.

### Source population

Dyads defined as all mothers to children under 5 years of age living in rural district of Enebsie Sarmidr in a 1 month period in 2016.

### Study population

Randomly selected cases and controls of Dyads who fulfill the inclusion criteria.

### Inclusion criteria

Dyads who lived at least 6 months in the study area.

### Exclusion criteria

Mothers unable to communicate due to illness or in voluntarism and controls from the same house of cases.

### Sample size

The sample size was calculated using Epi info 7 statcalc. The assumptions used for calculation were detecting a 3.5 times higher risk of suboptimal infant breast feeding practices among the cases and a 65.9 prevalence of suboptimal infant breast feeding practice among the controls based on the study done previously; 95% confidence interval (CI), 90% power and case to control ratio of 1:4 [7]. The case control ratio of 1: 4 was used to increase the power and sample size. As the number of cases were small, the sample size was increased by increasing the number of the controls. This made the minimum calculated sample size 58 for cases and 231 for controls. By taking 10% non-response rate the total sample size required was 318 having 64 cases and 254 controls.

### Sampling procedure

The district has 31 rural kebeles. All the 31 kebeles were included in the study. For this study, survey was conducted before the actual study and there were 13,122 under five children in the district. Of these, 115 were found with SAM. So the calculated sample size, 64 cases and 254 controls were selected in proportional allocation to the sample size of each of the 31 rural kebeles using computer generated simple random sampling technique in 1:4 case, control ratio. Totally 318 mother/care giver to child pairs were included.

### Variables

#### Dependent variables

- Severe acute malnutrition.

#### Independent variables

- **Socio demographic characteristics;** age of child, child sex, parental education, parental occupation, marital status of the mother, household economic status, maternal autonomy in decision making and family size
- **Nutrition and Child caring practices;** Exclusive breast feeding **(**EBF), Sub optimal EBF, late initiation of breast feeding.
- **Infection and childhood illness:** Fever, Human immune deficiency virus **(**HIV), diarrhea, other medical and surgical problems.
- **Obstetric History:** Antenatal care **(**ANC) visits, number of ANC visit, birth order, birth interval, use of extra food during pregnancy and lactation, number of children ever born.

### Operational definitions

#### Cases

“Dyads defined as all mothers to children under 5 years of age with SAM treated in an outpatient or inpatient therapeutic program in rural district of Enebsie Sarmidr in a 1 month period in 2016.

#### Controls

“Dyads defined as all mothers to children under 5 years of age with no diagnosis of severe acute malnutrition in rural district of Enebsie Sarmidr in a 1 month period in 2016.

#### Diarrhea

Unusual frequent and loose watery stool ≥3 times per day for 2 weeks or more.

#### Fever

Mothers/care takers thought as the child experienced unusual increase in temperature for 2 weeks or more.

#### Sub optimal exclusive breast feeding

Breast feeding less than eight times per day.

#### Household economic status

Monthly income of the family in Ethiopian Birr.

#### Maternal autonomy in decision making

Perceived maternal autonomy in decision making within the family.

#### Low income

Household monthly income less than 1000 Ethiopian Birr.

### Instrument and personnel

Interviewers followed semi structured questionnaires that were created from published research on the study area [4, 6, 8, 10]. Data collectors were three clinical nurses who took training on nutrition courses. Two clinicians supervised the data collection process. Two days of training was provided by the principal investigators to the data collectors & supervisors.

### Data quality control

Before data collection the questionnaire was first prepared in English and translated to the local language Amharic and finally again back to English for consistency. The questionnaire was pre tested on 5% of actual respondents in another kebele out of the study area. During the data collection time, regular monitoring and supervision of the overall activity was done by the supervisors and principal investigators to ensure the quality of data. All the collected data were checked, cleaned and coded to avoid some inconsistencies and incompleteness before analysis. Incomplete and inconsistent data were excluded from the analysis.

### Data processing and analysis

The data was cleaned, coded and entered in Epi data version 3.1 and transferred to statistical package for Social science (SPSS) version 20.0 for analysis. Emergency nutrition assessment (ENA) software was used to calculate anthropometric measurements. Both bivariate and multivariate logistic regressions were used to determine the association. Variables with (*P* < 0.2) in the bivariate logistic regression was included in the final model/multivariable. Statistical significance was made at 95% CI and *P*-value < 0.05 for multivariate analysis. The strength of association between independent and dependent variables was assessed using odds ratio with 95% confidence interval.

### Ethical issues

Ethical clearance was obtained from research and publication committee of Debre Markos University, College of Health Sciences. Informed written consent was obtained from the mothers.

## Results

The response rate was 97.8% (64 cases and 247 controls). The median age of the child in the Dyad, both cases and controls, was 18 months with standard deviation (SD) of 10.38 and 14.02 months respectively. The majority (71.9%) of children in the cases and more than half (56.3%) of the controls were between 7 and 24 months (Table 1).

**Table 1:** Sociodemographic characteristics of Mother/care giver with their under five children in rural district of Enebsie Sarmidr, March, 2016 (*N* = 311)

Variables		SAM status		Total N (%)
		Cases n (%)	Control n (%)	
Age of child	Birth −6 months	3(4.7%)	24(9.7%)	27(8.7%)
	7–24 months	46(71.9%)	139(56.3%)	185(59.5%)
	25–59 months	15(23.4%)	84(34.0%)	99(31.8%)
	Total	64(100.0%)	247(100.0%)	311(100.0%)
Sex of children	Male	32(50.0%)	118(47.8%)	150(48.2%)
	Female	32(50.0%)	129(52.2%)	161(51.8%)
	Total	64(100.0%)	247(100.0%)	311(100.0%)
Family size	≤5 family members	22(34.4%)	130(52.6%)	152(48.9%)
	≥6 family members	42(65.6%)	117(47.4%)	159(51.1%)
	Total	64(100.0%)	247(100.0%)	311(100.0%)
Family average income ETB	≤500	48(75.0%)	167(67.6%)	215(69.1%)
	(500–999]	14(21.9%)	60(24.3%)	74(23.8%)
	≥1000	2(3.1%)	20(8.1%)	22(7.1%)
	Total	64(100.0%)	247(100.0%)	311(100.0%)
The authority of decision making in the family	Mother	4(6.2%)	19(7.7%)	23(7.4%)
	Father	22(34.4%)	89(36.0%)	111(35.7%)
	Both father & mother	38(59.4%)	139(56.3%)	177(56.9%)
	Total	64(100.0%)	247(100.0%)	311(100.0%)
Respondents’/ Care givers’ religion	Orthodox	59(92.2%)	238(96.4%)	297(95.5%)
	Muslim	5(7.8%)	9(3.6%)	14(4.5%)
	Total	64(100.0%)	247(100.0%)	311(100.0%)
Marital status	Married & Lives with husband	57(89.1%)	221(89.5%)	278(89.4%)
	Lives without husband	7(10.9%)	26(10.5%)	33(10.6%)
	Total	64(100.0)	247(100.0)	311(100.0)

About (87.5%) of the cases and (97.2%) of the controls were born from mothers who had ANC follow up. There was similar equivalency (53.1% cases to 58.3%) of mothers who were seen 1–3 times in ANC follow up (Table 2).

**Table 2:** Obstetric history of mothers/care taker with their under five children and SAM status at rural district of Enebsie Sarmidr March, 2016 (*N* = 311)

Variables		SAM status		Total N (%)
		Cases n (%)	Controls n (%)	
Number of children ever borne	≥ 4 Children	20(31.3%)	83(33.6%)	103(33.1%)
	1–3 children	41(64.1%)	162(65.6%)	203(65.3%)
	I do not remember	3(4.7%)	2(0.8%)	5(1.6%)
	Total	64(100.0%)	247(100.0%)	311(100.0%)
ANC follow up	Yes	56(87.5%)	240(97.2%)	296(95.2%)
	No	8(12.5%)	7(2.8%)	15(4.8%)
	Total	64(100.0%)	247(100.0%)	311(100.0%)
Number of ANC follow up	≥4 times	27(42.2%)	90(36.4%)	117(37.6%)
	1–3 times	34(53.1%)	144(58.3%)	178(57.2%)
	I do not remember	3(4.7%)	13(5.3%)	16(5.1%)
	Total	64(100.0%)	247(100.0%)	311(100.0%)
Feeding status during pregnancy and lactating	extra meal/day	37(57.8%)	225(91.1%)	262(84.2%)
	No difference/reduced my meal	27(42.2%)	22(8.9%)	49(15.8%)
	Total	64(100.0%)	247(100.0%)	311(100.0%)
Reason for increasing feeding	Health workers thought and clear	37(100.0%)	221(98.2%)	259(98.5%)
	Any other reason	0(0.0%)	4(1.8%)	4(1.5%)
	Total	37(100.0%)	225(100.0%)	262(100.0%)
Reasons for decreasing feeding during pregnancy	Believing not be comfortable for fetus	24(88.9%)	16(72.7%)	40(81.6%)
	Believing fetal growth would be facilitated and delivery complicated	2(7.4%)	5(22.7%)	7(14.3%)
	Others	1(3.7%)	1(4.5%)	2(4.1%)
	Total	27(100.0%)	22(100.0%)	49(100.0%)
Birth interval	Less than 2 years	24(37.5%)	52(21.1%)	76(24.4%)
	2 and above years	40(62.5%)	195(78.9%)	235(75.6%)
	Total	64(100.0%)	247(100.0%)	311(100.0%)

Approximately two-thirds (70.3%) of the cases and almost all (91.1%) of the controls had initiated breast feeding within an hour of birth. A similar statistical difference (78.1% of the cases compared to 96.8%of the controls) was found related to exclusive breast feeding for the 6 months of life (Table 3).

**Table 3:** Child nutrition and care practices of under five children and their SAM status at rural district of Enebsie Sarmidr district, March, 2016, (*N* = 311)

Variables		SAM status		Total N (%)
		Cases n (%)	Controls n (%)	
Breast feeding (BF) Initiation within an hour after delivery	Yes	45(70.3%)	225(91.1%)	270(86.8%)
	No	19(29.7%)	22(8.9%)	41(13.2%)
	Total	64(100.0%)	247(100.0%)	311(100.0%)
EBF for the first 6 months	Yes	50(78.1%)	239(96.8%)	289(92.9%)
	No	14(21.9%)	8(3.2%)	22(7.1%)
	Total	64(100.0%)	247(100.0%)	311(100.0%)
Optimum breast feeding(≥8times/day)	Yes	28(43.8%)	231(93.5%)	259(83.3%)
	No	36(56.2%)	16(6.5%)	52(16.7%)
	Total	64(100.0%)	247(100.0%)	311(100.0%)
Total time of breast feeding	1–24 months	5(7.8%)	17(6.9%)	22(7.1%)
	≥25 months	59(92.2%)	230(93.1%)	289(92.9%)
	Total	64(100.0%)	247(100.0%)	311(100.0%)
History of prelacteal feeding	Yes	13(20.3%)	21(8.5%)	34(10.9%)
	No	51(79.7%)	226(91.5%)	277(89.1%)
	Total	64(100.0%)	247(100.0%)	311(100.0%)

### Determinants of severe acute malnutrition

Variables that showed an association with the outcome variable at *P* value (*P* < 0.2) in the bivariable analysis were entered into the final multivariable analysis. Odds ratio along with 95% confidence interval was estimated to assess the strength of the association and P value < 0.05 was used to declare the statistical significance in the multivariable analysis.

Sociodemographic determinants; age group below 2 years [Crude odds ratio (COR) = 1.683, 95% CI, (0.892–3.178)], family size who had 6 and above (COR = 0.47, 95% CI (0.26–0.87)), family income who had averagely less than 1000 Ethiopian Birr (COR = 2.731, 95%CI (0.621–12.004) had *P*-value less than 0.2 (Table 4).

**Table 4:** Bivariable analysis of determinants of SAM on under five children at rural kebeles of Enebsie Sarmidr district, March, 2016, (*N* = 311)

Variables		SAM status		COR (95%, CI)	*P*-value
		Cases n (%)	Controls n (%)		
Age	0–24 months	49(76.6%)	163(66%)	1.68 (0.89–3.17)	0.108
	25–59 months	15(23.4%)	84(34%)	1	
Family size	< 6	22(34.4%)	130(52.6%)	1	
	≥6	42(65.6%)	117(47.4%)	0.47(0.26–0.87)	0.10
Family income	< 1000 EBR	62(96.9%)	227(92%)	2.73(0.62–12.00)	0.183
	≥1000 EBR	2(3.1%)	20(8%)	1	
Prelacteal feeding	Yes	13(20.3%)	21(8.5%)	2.74(1.28–5.83)	0.009
	No	51(79.7%)	226(91.5%)	1	
Exclusive BF	Yes	50(78.1%)	239(96.8%)	1	
	No	14(21.9%)	8((3.2%)	8.36(3.33–21.00)	0.001
BF initiation within one hr. after delivery	Yes	45(70.3%)	225(91.1%)	1	
	No	19(29.7%)	22(8.9%)	4.318(2.16–8-62)	0.001
Diarrhea in the preceding 2 weeks before developing SAM	Yes	14(21.9%)	9(3.6%)	7.40(3.03–18.05)	0.001
	No	50(78.1%)	238(96.4%)	1	
Febrile illness in the preceding 2 weeks before developing SAM	Yes	13(20.3%)	20(8.1%)	2.89(1.351–6.19)	0.006
	No	51(79.7%)	227(91.9%)	1	
Having ANC follow up	Yes	56(87.5%)	240(97.2%)	1	
	No	8(12.5%)	7(2.8%)	4.89(1.70–14.07)	0.003
Feeding status during pregnancy	I ate least one extra mealing	37(57.8%)	225(91.1%)	1	
	Did not increase or decreased mealing	27(42.2%)	22(8.9%)	7.46(3.851–14.46)	0.001
Birth interval	< 2 years	24(37.5%)	52(21.1%)	2.25(1.24–4.06)	0.007
	> = 2 years	40(62.5%)	195(78.9%)	1	

In multivariate analysis, children’s aged between birth to 24 months were 2.6 times more likely to be severely acute malnourished than those children aged between 25 and 59 months [adjusted odds ratio (AOR) = 2.63, 95% CI 1.17–5.95]. Mothers who did not initiate breast feeding within 1 h after delivery were more than 4 times more likely to have severely acute malnourished child than mothers’ who initiated to fed their breast within 1 h after delivery(AOR = 4.25,95% CI 1.74–10.41). Likewise children who were not fed breast milk exclusively for their first 6 months of age were 5.8 times more likely to develop severe acute malnutrition than those who were fed exclusively (AOR =5.81, 95% CI 1.79–18.78).

Severe wasting was 7.9 times more likely to occur in children who had diarrhea within 2 weeks before developing SAM than those children who did not have diarrhea within 2 weeks before developing SAM (AOR = 7.97, 95% CI 2.57–24.73. Likewise severe acute malnutrition was 2.8 times more likely to occur in children who had febrile illness within 2 weeks before developing SAM than those children who did not have febrile illness within 2 weeks before developing SAM (AOR = 2.87 95% CI 1.13–7.62).

Similarly children who were born from mothers who didn’t change their feeding status from the regular level during pregnancy and lactation were 8 times more likely to be severely acute malnourished than those who were born from mothers who had increased at least one extra meal per day during pregnancy and lactation (AOR = 8.15, 95% CI 3.69–17.97). Similarly children who were born from mothers with birth interval of less than 2 years were more than 3 times highly exposed to severe acute malnutrition than those who were born from mothers who had ≥2 years of birth interval (AOR = 3.33, 95% CI 1.54–7.19) (Table 5).

**Table 5:** Multivariable analysis of determinants of SAM among under five children at rural kebeles of Enebsie Sarmidr district, March, 2016, (*N* = 311)

Variables		Nutritional status		AOR (95% CI)	*P*-value
		Cases n (%)	Controls n (%)		
Age	Birth −24 months	49(76.6%)	163(66%)	2.637(1.1691–5.950)	0.019
	25–59 months	15(23.4%)	84(34%)	1	
BF initiation within one hr. After delivery	Yes	45(70.3%)	225(91.1%)	1	
	No	19(29.7%)	22(8.9%)	4.257(1.740–10.416)	0.002
Exclusive breast feeing	Yes	50(78.1%)	239(96.8%)	1	
	No	14(21.9%)	8((3.2%)	5.812(1.798–18.781)	0.003
Febrile illness preceding 2 weeks before developing SAM	Yes	13(20.3%)	20(8.1%)	2.870(1.133–7.628)	0.026
	No	51(79.7%)	227(91.9%)	1	
Diarrhea preceding 2 weeks before developing SAM	Yes	14(21.9%)	9(3.6%)	7.977(2.572–24.739)	0.001
	No	50(78.1%)	238(96.4%)	1	
Feeding practice during pregnancy	Increased at least one additional	37(57.8%)	225(91.1%)	1	
	Remained as regular/decreased from the regular	27(42.8%)	22(8.9%)	8.151(3.696–17.976)	0.001
Birth interval	< 2 years	24(37.5%)	52(21.1%)	3.337(1.547–7.199)	0.002
	≥2 years	40(62.5%)	195(78.9%)	1	

## Discussion

In this study, children’s age group of birth-24 months showed significant association with severe acute malnutrition. The result is similar to studies done in Ethiopia, India, Katranaka [11, 12].

Those who are in age groups (≤24 months) were three times more likely to develop SAM than their counter parts. This might from because the first 2 years of life are extremely important for the growth and development of children. And it is a time for most malnutrition cases to occur because it is a transition time from exclusive breast feeding to complementary feeding.

Maternal initiation of breast feeding after 1 h of delivery was significantly associated with severe acute malnutrition. Children who were breast fed within the first hour after birth were four times more likely to develop severe acute malnutrition when compared to those who initiated breast feeding within an hour of birth. The result is in line with a study done in Kenya where non-exclusive breast feeding was significantly associated with severe acute malnutrition and wasting. Babies not exclusively breast fed in the Kenya study were 6 times more likely to develop wasting and severe acute malnutrition [10]. Additional studies supported by the result from this study [5, 6, 8, 13]. This fact, that breast milk contains everything the baby wants for the first 6 months of life and exclusive breast feeding, supports the role of breast milk in assisting the infant fight infections including diarrheal diseases which may later result in malnutrition.

Diarrhea within 2 weeks before the onset of SAM was significantly associated with severe acute malnutrition. Those children who had diarrheal diseases were eight times more likely to develop SAM when compared to their counterparts. Similar findings were found in the studies done in Bhandari hospital, Mongo district, EDHS report and Felege Hiwot [4, 9, 11, 14]. This might from the fact that diarrheal diseases may result in loss of body fluids and electrolyte and nutritional imbalance.

Similarly febrile illness within 2 weeks before the onset of SAM was significantly associated with severe acute malnutrition. Those who had febrile illness within 2 weeks before SAM were three times more likely to develop SAM than those who hadn’t febrile illness. The result is in line with other findings [8, 10].

Children who were born from mothers with narrow birth interval (< 2 years) were three times more likely to develop severe acute malnutrition. The result is similar with the studies done in East Ethiopia [7, 10, 13, 15]. This might from the fact that decreased birth interval increases the number of children which may in turn affect child feeding practice.

Children who were born from mothers who didn’t increase feeding during pregnancy and/or lactation were eight times more likely to develop SAM than others. The result is not in congruent with a study done at Dollo Ado where the risk of being wasted was 1.55 times higher among mother of children who consumed extra food during pregnancy than previous [16]. This might from the fact that the fetus during pregnancy and the infant afterwards acquires its nutrition from the mother as a result mothers need extra food during pregnancy and breast feeding.

## Conclusions

Age of the child, initiation of breast feeding 1 h after delivery, nonexclusive breast feeding, diarrheal disease and febrile illnesses within 2 weeks before SAM, decreased or no change of mealing compared to the regular during pregnancy and lactating of the mother and narrow birth interval were determinants of SAM.

As a result, collaborative efforts should be organized to improve promotion of better child caring practices through appropriate age specific child and maternal feeding practices, prevention and early treatment of acute childhood illnesses and promotion of birth spacing through family planning.

## Abbreviations

ANC: Ante natal care
AOR: Adjusted odds ratio
BF: Breast feeding
CI: Confidence interval
COR: Crude odds ratio
EBF: Exclusive breast feeding
EDHS: Ethiopia demographic and health survey
HIV: Human immune deficiency virus
Km: Kilometer
SAM: Sever acute malnutrition
SD: Standard deviation
SPSS: Statistical package for social science
